# The choice between a ritonavir-boosted protease inhibitor- and a non-nucleoside reverse transcriptase inhibitor-based regimen for initiation of antiretroviral treatment – results from an observational study in Germany

**DOI:** 10.1186/s40545-016-0092-4

**Published:** 2016-12-30

**Authors:** Jörg Mahlich, Mona Groß, Alexander Kuhlmann, Johannes Bogner, Hans Heiken, Matthias Stoll

**Affiliations:** 1 Janssen KK, Health Economics, Tokyo, Japan; 2 Heinrich-Heine University of Düsseldorf, Düsseldorf Institute for Competition Economics (DICE), Düsseldorf, Germany; 3 University of Hanover, Centre for Health Economics Research, Hanover, Germany; 4 Division of Infectious Diseases, Ludwig Maximilian University of Munich, Munich, Germany; 5 Private Practice, Hanover, Germany; 6 Hannover Medical School (MHH), Centre for Internal Medicine, Hanover, Germany

**Keywords:** HIV infection, AIDS, NNRTI, PI, Antiretroviral treatment, Treatment decision

## Abstract

**Background:**

This study aims at identifying predictors of the treatment decision of German physicians with regard to a non-nucleoside reverse transcriptase inhibitor (NNRTI) or a ritonavir-boosted protease inhibitor (PI/r) -based initial treatment regimen.

**Methods:**

The study is based on a sub analysis of a nation-wide multi-centre, non-interventional, prospective cohort study. 133 patients were identified, who received antiretroviral first-line therapy. By means of a logistic regression, factors that determine the treatment strategy for treatment-naïve patients were analysed.

**Results:**

Compared to patients receiving a NNRTI-based initial regimen, patients treated with PI/r are slightly younger, less educated, in a later stage of HIV and have more concomitant diseases. Regression analysis revealed that being in a later stage of HIV (CDC-C) is significantly associated with a PI/r-based treatment decision.

**Conclusions:**

Our analysis is the first study in Germany investigating sociodemographic and disease-specific parameters associated with a NNRTI- or a PI/r-based initial treatment decision. The results confirm that the treatment decision for a PI/r strategy is associated with disease severity.

## Background

Since combination antiretroviral therapy (cART) became available in 1996, progression to an immunodeficient state of HIV infection can be effectively avoided, reducing both mortality and morbidity associated with HIV infection [1, 2]. In addition, by the effective reduction of viral load, cART is able to reduce the risk of horizontal and vertical HIV transmission [3].

Actual guidelines for the initiation of cART in HIV-infected individuals recommend antiretroviral regimens consisting mainly of two nucleoside reverse transcriptase inhibitors (nRTI) combined with either a non-nucleoside reverse transcriptase inhibitor (NNRTI) or a ritonavir-boosted protease inhibitor (PI/r) or, more recently, an integrase strand transfer inhibitor (INSTI) [4, 5]. While a recent meta-analysis did not find differences between NNRTI- and PI/r-based regimens in terms of clinical outcomes such as death or progression to AIDS, it was found that trial-defined virological failure was higher with a NNRTI-based regimen [6]. Also, toxicity of certain NNRTIs were found to be inferior to PI/r-based regimen [7]. Hence, the 2015 updates of both the national German guidelines as well as the US DHHS-guidelines do not longer recommend NNRTI based regimens as an preferred or unrestricted option for the initiation of cART [4, 5].

Important predictors of virological failure are antiretroviral resistance and inadequate antiretroviral medication adherence to cART [8, 9]. Both factors are linked in that inadequate adherence increases the risk of emerging resistance against antiretroviral drugs [10] by allowing HIV replication in the presence of sub-therapeutic drug levels. Several factors, in turn, have been reported to negatively influence treatment adherence: sociodemographic factors such as younger age [11, 12], lower income [13], unemployment [14], and low educational level [15]; treatment-related factors such as high intake frequency [15, 16]; disease-specific factors such as advanced disease stage [17], occurrence of adverse events [18], or the number of concomitant diseases [19]; and physician-patient relationship factors such as lack of mutual trust between the physician and the patient [11, 16]. Factors related to clinical setting such as insufficient medical health care [13] or the absence of patient education programs [20] have been identified as well.

Further results indicate that an impaired adherence influences medication resistance more intensively in NNRTI-based than in PI/r-based strategies [8, 21, 22]. Moreover, NNRTI-associated resistance is associated with an elevated morbidity and mortality [23, 24]. As a consequence, it is assumed that people living with HIV or AIDS (PLWHA) who do not properly adhere to their medication are more likely to receive a PI/r- rather than a NNRTI-based treatment regimen by their physicians in order to prevent potential damages. Our analysis intends to analyze if this conjecture holds true for the case of German HIV specialists. Based on data of the German ‘Cost and Resource Utilisation Study in Antiretroviral Therapy’ (CORSAR) [25], we analyze parameters that influence PLWHA’s adherence to identify potential predictors of the treatment decision with regard to a NNRTI- or a PI/r-based initial treatment regimen. To our knowledge, this is the first study that explores this topic in Germany, while earlier work focused on Switzerland [26] and the UK [27]. As both treatment guidelines and the costs of medication differ significantly between the countries, institutional differences of the respective health care delivery systems should be taken into account. In Germany, for instance, the average annual medical costs for a NNRTI-based regimen was 25,058 Euros, while it was 38,334 Euros for a PI/r-based regimen [28]. The high rate of medical innovation in this indication further underlines the need for new data that lead to a better understanding of factors for treatment decision.

## Methods

The analysis is based on the “Cost and Resource Utilisation Study in Antiretroviral Therapy” (CORSAR), a Germany-wide, multi-centre, non-interventional, prospective cohort study to evaluate costs of HIV disease in cART-treated PLWHA [22]. Inclusion criteria for CORSAR were confirmed HIV diagnosis, age of at least 18 years, cART at study entry, and written informed consent. The observation period was 96 weeks between April 2009 (first patient started) and April 2012 (last patient finished), with scheduled quarterly visits to the involved physician. The CORSAR project was not meant to include primarily treatment-naïve patients. All participants from eight private practices specialized in HIV and four hospitals went through a six-month period with baseline examinations. The CORSAR data set included 1154 PLWHA representing a 2.3% sample of treated PLWHA in Germany [29, 30]. 11.5% (*n* = 133) of the study population was treatment-naïve and had been included contemporaneously with treatment start between April 2009 and May 2010. The CORSAR survey was approved by the national regulatory authorities and local ethics committees of all participating centers. All patients were given thorough information on the survey. Written informed consent was obtained from all study participants.

Our analysis is based on the treatment naive subgroup, which represents a sample of patients in a recent and real clinical life setting to analyse parameters determining the choice of a particular cART regimen. Baseline data variables that are used for the analysis are reported in Table 1.

**Table 1:** Variables

Treatment regimen	• NNRTI-based • PI/r-based • Other
Sociodemographic factors	
Patient age (in years) at diagnosis	• < 50 years old
	• 50 years or older
Education level	• With college degree
	• Without college degree
Anamnestic factors	
HIV stage according to CDC classification	• A (asymptomatic)
	• B (symptomatic)
	• C (AIDS-defining diseases)
More than three concomitant diseases	• No
	• Yes

To evaluate parameters corresponding with the treatment decision, three therapy groups were established: (1) NNRTI-based initial treatment regimen consisted of one NNRTI, such as Efavirenz or Rilpivirine, in addition to nucleos(t)ide analogues but no PI; (2) PI-based initial treatment regimen consisted of one ritonavir boosted PI such as Atazanavir or Darunavir in addition to nucleos(t)ide analogues; and (3) other initial treatment regimen such as integrase inhibitors, entry (CCR5)-inhibitors, or nuke-sparing regimens (e.g. boosted double-PI/r therapy). During the study-period the use of INSTI was less common than in the time since 2014. As the category “other” is rather small and quite heterogeneous, the focus of our analysis lies on the choice between a NNRTI- and PI/r-based regimen. While most of the variables are self-explanatory, the CDC classification might need some additional explanation. Clinical staging of HIV disease is performed in accordance with the CDC classification for HIV infection [31]. By definition of the CDC classification, the staging reflects the most advanced stage of disease and does not allow a reassignment to a less advanced stage, not even in cases with a complete and stable recovery from immunodeficiency and AIDS-defining diseases under cART.

Due to the small sample size and the limited degrees of freedom, we decided to merge the HIV stages A and B to come up with only two categories (non-AIDS vs. AIDS stage).

We employed a logistic regression to identify factors that determine the choice of the treatment strategy. Descriptive statistics and regression analysis were performed using SPSS, Chicago; and STATA, College Park software respectively. Based on the findings of earlier studies on adherence that were touched upon in the introduction, we expect that physicians assume patients who are younger, already in a progressed stage of HIV, and have concomitant diseases to be less adherent to their medication. Consequently, patients with those characteristics may be prone to receive a PI/r-based treatment regimen because impaired adherence influences medication resistance more intensively in NNRTI-based than in PI/r-based strategies.

## Results

Patient characteristics according to the treatment regimen are reported in Table 2. Compared to patients receiving NNRTI, PI/r-treated patients are slightly younger, less educated, in a later stage of HIV and have more concomitant diseases. For instance, only 11% of the patients who receive an NNRTI-based treatment regimen are in disease stage C, while the large majority is in either disease state A or B. On the other hand, 37% of the patients with a PI/r-based treatment regimen have progressed already into disease stage C. The results are in line with the expectations, as all three variables are frequently associated with poor adherence.

**Table 2:** Patient characteristics

		NNRTI	PI	Other	Total
		(*n* = 46) [34.6%]	(*n* = 38) [28.6%]	(*n* = 49) [36.8%]	(*N* = 133)
Sociodemographic factors					
Patient age (in years) at diagnosis	<50	36 [78.3%]	34 [89.5%]	43 [87.8%]	113 [85.0%]
	≥50	10 [21.7%]	4 [10.5%]	6 [12.2%]	20 [15.0%]
Education level (college degree)	No	36 [78.3%]	31 [81.6%]	41 [83.7%]	108 [81.2%]
	Yes	10 [21.7%]	7 [18.4%]	8 [16.3%]	25 [18.8%]
Anamnestic factors					
HIV stage according to CDC classification	A + B	41 [89.1%]	24 [63.2%]	36 [73.5%]	101 [75.9%]
	C (AIDS)	5 [10.9%]	14 [36.8%]	13 [26.5%]	32 [24.1%]
Three or more concomitant diseases	No	37 [80.4%]	29 [76.3%]	39 [79.6%]	105 [78.9%]
	Yes	9 [19.6%]	9 [23.7%]	10 [20.4%]	28 [21.1%]

The results of the multinomial logistic regression are reported in Table 3. The multivariate regression analysis confirms that NNRTI and PI treatment groups differ significantly with regard to the disease stage. Specifically, being in advanced HIV stage C significantly increases the likelihood of a PI/r-based regimen for initiation of antiretroviral treatment. This variable is also slightly significant when NNRTI and “other” treatment regimen are compared, while PI/r and “other” treatment regimen do not differ significantly and can be considered as interchangeable. All other variables were not significant due to the limited number of patients in our analysis.

**Table 3:** Multinomial logistic regression

Variable	Odds ratio		
	PI/r vs NNRTI	PI/r vs other	NNRTI vs other
50 years or older	0.606419	0.9194778	1.718239
	(0.463)	(0.904)	(0.363)
College degree	0.9528552	1.187664	1.38485
	(0.935)	(0.768)	(0.557)
AIDS	4.237344**	1.578817	0.3524308*
	(0.017)	(0.337)	(0.073)
3 or more concomitant diseases	0.8452713	1.20924	0.8452713
	(0.817)	(0.723)	(0.759)
Constant	0.6384591	0.6311049	1.004598
	(0.158)	(0.152)	(0.987)
N	84	87	95

*P* value in parentheses. * *p* < 0.10, ** *p* < 0.05

## Discussion

Our results confirm earlier research that observes a relatively low proportion of patients with a NNRTI-based first-line cART in Germany in comparison to other countries [32], although most NNRTI-based treatment regimens are significantly cheaper than PI/r-based ones. From the 133 patients receiving antiretroviral first-line therapy, only 34.6% received a NNRTI-based cART regimen. This is in contrast to countries like the UK, where almost 75% of first-line patients are on a NNRTI-based regimen [27]. Moreover, a survey of clinical practice in Europe found that the NNRTI Efavirenz is mandatory for first-line therapy in 71.4% of northern European clinics [33]. The early use of PI/r for treatment-naïve patients might be a specific feature of the German health care system. German guidelines are relatively liberal, as they recommend a broad spectrum of both PI/r- and NNRTI-based cART regimens as equivalent preferred options at the time of our study [4]. Additionally, there are fewer restrictions in Germany to choose equivalent, but more expensive cART regimens individually, if this is requested either by the treating physician or by the patient. Discrete choice-based preference studies demonstrated that the highest relevance for the treatment selection of German physicians, as well as patients, was the emotional quality of life indicated by the fact that the disease was not obvious for other persons, as stigma is probably still an issue in Germany [34, 35]. Those results show that German physicians also take treatment goals other than efficacy into account, which also explains the high variation in initial treatment regimens.

When it comes to the determinants of the treatment strategy, our results are in line with previous findings as well. A large British study, for instance, found clinic site and a prior AIDS diagnosis (CDC stage C) were the most important factors associated with use of a PI/r-based initial treatment regimen [27]. Their findings indicate that in the early days of cART, the prescription of a PI/r-based regimen was more likely. More recently, however, NNRTIs are on the rise mainly due to increased cost considerations. In the framework of a multivariate analysis of a Swiss cohort it was also found that compared with an Efavirenz (NNRTI) -based initial treatment, starting with the protease inhibitor Lopinavir/r was associated with prior AIDS and higher viral load [26].

Other factors that have been identified as predictors of treatment adherence such as the number of concomitant diseases [19] are not associated with the treatment decision in our data, possibly due to our small sample size. Another possible explanation is that physicians either consider predictors of adherence other than the ones identified in the empirical literature, or they prescribe PI/r for reasons other than preventing drug resistance caused by poor adherence. Alternatively, HIV physicians are not able to predict the prospective adherence of their patients. It was found that clinicians tend to overestimate the actual adherence of their patients [36, 37] when they were asked for a retrospective assessment. While suggestions have been put forward to improve the assessment of adherence by using the data of viral load and HIV genome sequence to identify non-adherence amongst patients under treatment [38], things get much more difficult when it comes to the *a priori* assessment of the potential adherence of previously untreated patients when initializing cART. Given the high uncertainty about potential adherence of patients and the relatively small sample size, we consider the significant impact of the disease stage on the treatment decision as a strong result. In fact, a recent German data analysis confirmed our results with a much broader sample [39].

The limitation of the use of our data is the small sample size. Moreover, in the meantime, new treatment options have been introduced in first-line recommendations such as integrase inhibitors. This may have altered the use of cART in treatment-naïve subjects after the termination of our study. Here would seem the logical starting point for a future study.

## Conclusions

Our data reflect real clinical practice of German physicians on parameters corresponding with treatment decisions for antiretroviral-naïve patients with regard to the use of NNRTI or PI/r in the years 2009 to 2012. Our analysis is the first study in Germany investigating sociodemographic and disease-specific parameters associated with a NNRTI- or a PI/r-based initial treatment decision. The results confirm that the treatment decision for a PI/r strategy is associated with disease severity. However, future research should analyse further baseline parameters in more detail. Also drug- and physician-specific variables such as physician training should be taken into account to better understand the underlining mechanism of prescription behaviour.

## Abbreviations

cART: Combination antiretroviral therapy
CDC: Centers for Disease Control and Prevention classification system
CORSAR: Cost and Resource Utilization Study in Antiretroviral Therapy
INSTI: Integrase strand transfer inhibitor
NNRTI: Non-nucleoside reverse transcriptase inhibitor
nRTI: Nucleoside reverse transcriptase inhibitor
PI/r: Ritonavir-boosted protease inhibitor
PLWHA: People living with HIV or AIDS
